# Genetic diversity and clinical spectrum of *Mycobacterium abscessus* complex in India

**DOI:** 10.1016/j.ijregi.2026.100955

**Published:** 2026-07-22

**Authors:** Vignesh Balaji, Marilyn Mary Ninan, Suchitha Chase, Richa Gupta, Joy Sarojini Michael

**Affiliations:** 1 Department of Clinical Microbiology, Christian Medical College, Vellore, India; 2 The Tamil Nadu Dr. M.G.R. Medical University, Chennai, India; 3 Department of Surgery Unit-III, Christian Medical College, Vellore, India; 4 Department of Respiratory Medicine, Christian Medical College, Vellore, India

**Keywords:** Mycobacterium abscessus complex, Multilocus sequence typing (MLST), Whole-genome sequencing (WGS), Molecular epidemiology, Sequence types (STs)

## Abstract

**Objectives:**

The *Mycobacterium abscessus* complex is a rapidly growing nontuberculous mycobacterium that causes chronic lung, skin, and soft-tissue infections. Disease management is challenging because of prolonged antibiotic therapy and resistance, making knowledge of local strain diversity and resistance patterns essential. However, the molecular epidemiology of *M. abscessus* in India remains largely unreported.

**Methods:**

Whole-genome sequencing and multilocus sequence typing (MLST) were performed on 50 clinical isolates collected from patients across 10 Indian states (2019-2024). Genomic DNA was extracted, and sequencing was performed using the Illumina platform. MLST was conducted according to the PubMLST scheme based on seven housekeeping genes.

**Results:**

Analysis of the 50 isolates from 10 states across India showed a predominance of *M. abscessus* subsp. *abscessus* (76%), with extrapulmonary infections, particularly skin and soft-tissue infections. No isolates of subsp. *bolletii* were detected. MLST revealed genetic heterogeneity. *M. abscessus* subsp. *abscessus* isolates were distributed across 8 known and 18 novel sequence types (STs), with ST-5 being the most common, while *M. abscessus* subsp. *massiliense* isolates were predominantly assigned to ST-46, ST-33, and ST-37, along with two novel STs. Overall, 40% of the isolates represented novel STs.

**Conclusion:**

This study revealed extensive genomic diversity, with 40% of the isolates representing novel STs. The predominance of subsp. *abscessus* and the high frequency of extrapulmonary infections highlight region-specific trends and underscore the need for strengthened genomic surveillance.

## Highlights

• Multicenter multilocus sequence typing of 50 *Mycobacterium abscessus* isolates from 10 different Indian states.
• Forty percent of the isolates represented novel sequence types.
• Predominance of *Mycobacterium abscessus* subsp. *abscessus* (76%), with frequent extrapulmonary infections.
• Novel sequence types were associated with subspecies-specific clinical patterns.
• This study provides the first comprehensive genomic and clinical characterization of *M. abscessus* in India.

## Introduction

Nontuberculous mycobacteria (NTM) are ubiquitous environmental pathogens associated with a broad spectrum of pulmonary and extrapulmonary infections. Worldwide, the increasing incidence observed over the past two decades has been attributed to advances in diagnostic techniques and a growing population of immunocompromised individuals. Their clinical presentation often mimics that of *Mycobacterium tuberculosis*, resulting in diagnostic errors and suboptimal treatment [1,2]. Among the NTMs, the *Mycobacterium abscessus* complex is a leading cause of pulmonary disease among patients with cystic fibrosis (CF) [3], as well as postsurgical infections in immunocompetent patients. The *Mycobacterium abscessus* complex (MABC) comprises three subspecies: *M. abscessus* subsp. *abscessus, massiliense*, and *bolletii,* with distinct genomic and phenotypic characteristics [4]. Accurate subspecies identification is essential, as clinical outcomes and antimicrobial susceptibility vary because of differences in inducible macrolide resistance mediated by the *erm(41)* gene [5]. *M. abscessus* infections require prolonged antibiotic therapy because the organism exhibits high intrinsic and inducible resistance, particularly to macrolides. Current multidrug regimens achieve culture conversion in <50% of patients, with many experiencing persistent infection, relapse, or progression to fatal outcomes. In the absence of treatment, these infections can lead to mortality and progressive lung damage [6].

Genomic epidemiology data on non-CF populations remain limited, and emerging studies indicate that individual subspecies or strains are linked to particular patient populations and exhibit variable clinical outcomes. Understanding the genetic diversity of the *M. abscessus* complex is critical, as genomic variation affects virulence, antimicrobial susceptibility, and potential transmissibility, all of which have direct clinical implications [7]. Comprehensive knowledge of local strain diversity, resistance determinants, and associated clinical features is therefore crucial for effective patient management.

Prolonged *M. abscessus* infections can result in the emergence of diverse, resistant variants within different lung regions, which may be missed by single-sputum analyses, highlighting the need for longitudinal studies [8]. Genomic tools such as multilocus sequence typing (MLST) and whole-genome sequencing-based typing offer high discriminatory power to delineate circulating clones and unravel epidemiological patterns [9]. Recent genomic studies have further demonstrated that lineages within the *M. abscessus* complex differ widely in virulence, environmental survival, and antimicrobial resistance within the complex, driven by factors such as efflux pump modulation, mutations in cell wall–associated genes, and the presence of mobile genetic elements. Larger sequencing studies from Europe, the United States, and East Asia have identified the dominant clones that are present across clinical settings and are often linked to more severe disease, highlighting the importance of incorporating molecular epidemiology into routine diagnostic strategies [10].

In India, the true burden of *M. abscessus* infections remains poorly defined because of diagnostic challenges, limited availability of molecular identification tools, and frequent misclassification as tuberculosis [11]. Data integrating subspecies-level molecular epidemiology with detailed clinical features and treatment outcomes are scarce. Although clinically important, genomic surveillance of MABC in India remains limited, leaving critical gaps in understanding the strain diversity, resistance mechanisms, and their clinical implications [12]. This study, therefore, aims to comprehensively characterize the clinical manifestations, treatment practices, and outcomes of patients with MABC infections in India. It also aims to perform molecular epidemiological profiling of circulating isolates through subspecies identification and MLST.

## Methods

### *Strain selection and culture*

This retrospective study analyzed MABC isolates from 50 patients diagnosed with MABC infection at Christian Medical College (Vellore, India) between January 2019 and December 2024. Ethical approval was obtained from the institutional review board (IRB Min No: 15312). All isolates were processed in the National Tuberculosis Elimination Programme-accredited TB containment laboratory under negative pressure, with quality control performed according to standard protocols.

### *Isolation and identification*

#### Isolation

Clinical specimens were decontaminated using the *N*-acetyl-L-cysteine-NaOH method and cultured on both Mycobacterium growth indicator tube and Lowenstein-Jensen (LJ) medium. Isolates were confirmed as *M. abscessus* by Ziehl-Neelsen staining, biochemical tests, matrix-assisted laser desorption/ionization time-of-flight (MALDI-TOF), and were subsequently subcultured on LJ slants for analysis.

#### Identification

**Biochemical identification:** Arylsulfatase activity, 3% NaCl tolerance, and nitrate reduction were performed according to standard protocols. These biochemical markers cannot reliably distinguish subspecies, but they provide useful supportive evidence for MABC identification and remain valuable in settings where rapid, low-cost diagnostic approaches are needed.

**MALDI-TOF:** Well-isolated colonies of NTM from LJ medium were processed using the VITEK® MS Mycobacteria/Nocardia Kit (BioMérieux) for protein extraction, following the manufacturer’s protocol.

### *Whole-genome sequencing*

Genomic DNA was extracted using the QIAamp DNA Mini Kit and quantified using a Nanodrop™ 2000. Next-generation sequencing libraries were generated from the extracted genomic DNA using the Illumina KAPA HyperPrep Library Preparation Kit, following the manufacturer’s instructions. Libraries were sequenced in a 150-bp paired-end run on the Illumina Novaseq 6000 Platform (Illumina, San Diego, CA, USA). Raw reads were processed with TrimGalore (v0.6.10) and assembled using the SPAdes genome assembler [13].

### *Subspecies identification*

Average nucleotide identity values were calculated using the Pyani package (v0.2.12) (https://github.com/widdowquinn/pyani) with reference genomes for *M. abscessus* subsp. *abscessus* ATCC 19977 (GenBank: GCA_000069185.1), *M. abscessus* subsp. *bolletii* BD (GenBank: GCA_003609715.1), and *M. abscessus* subsp. *massiliense* str. GO 06 (GenBank: GCA_000277775.2).

### *MLST*

Typing was performed using the PubMLST scheme (seven genes) with the MLST software available at https://github.com/tseemann/mlst [14].

### *Phylogenetic construction*

MLST gene sequences were aligned using MUSCLE in MEGA (Molecular Evolutionary Genetics Analysis) software [15]. Phylogenetic relationships were constructed using the Neighbor-Joining method based on pairwise distances. Robustness was assessed by 1000 bootstrap replicates, with bootstrapped values ≥70% considered indicative of strong node support. The trees were exported in Newick format and visualized using the Interactive Tree of Life online platform, enabling enhanced graphical representation and metadata integration [16].

## Results

### Isolation and identification

A total of 50 MABC strains were isolated. Biochemical characterization was performed for all isolates using arylsulfatase activity, 3% NaCl tolerance, and the nitrate reduction assay. All isolates were positive for arylsulfatase activity and demonstrated tolerance to 3% NaCl, supporting their classification as rapidly growing mycobacteria. In addition, all isolates were positive for nitrate reduction, further supporting their identification as *Mycobacterium abscessus*. However, these biochemical markers were not sufficient to reliably distinguish subspecies within the complex.

All isolates were also subjected to MALDI-TOF MS analysis, which confirmed their identification as *Mycobacterium abscessus*. Subspecies-level classification was subsequently established using molecular methods.

### Subspecies distribution

Among the 50 MABC isolates, *M. abscessus* subsp. *abscessus* predominated, accounting for 76% (38/50) of the isolates, whereas *M. abscessus* subsp. *massiliense* accounted for 24% (12/50). *M. abscessus* subsp. *bolletii* was not identified in this study. Statistical testing was not performed because of the limited number of isolates.

### Geographic distribution

The patients originated from 10 states, with most from West Bengal (n = 16), Bihar (n = 9), and Tamil Nadu (n = 9). Patients with pulmonary *M. abscessus* infections were predominantly from Bihar (n = 5) and West Bengal (n = 4), whereas those with extrapulmonary infections were predominantly from West Bengal (n = 12) and Tamil Nadu (n = 6). Sequence type 5 (ST-5) was the most prevalent in this study and occurred most frequently in patients from West Bengal (n = 8) and Bihar (n = 3).

### *Clinical details*

**Age, gender, and occupation:** Most patients were aged 31-60 years, with a male predominance (27/50, 54.0%), which was consistent across both subspecies (*M. abscessus* subsp. *abscessus*: 20/38, 52.6%; *M. abscessus* subsp. *massiliense*: 7/12, 58.3%). Among the 23 female patients, 18 were infected with *M. abscessus* subsp. *abscessus* and five with *M. abscessus* subsp. *massiliense*. With respect to occupation, homemakers accounted for 15/50 (30%), followed by farmers (12%). Occupational details were unavailable for 12 patients. The demographic characteristics of the patients are summarized in Table 1.

**Table 1:** Demographic characteristics of patients with MABC infections.

Characteristics	*M. abscessus* (n = 38)	*M. massiliense* (n = 12)	Total (n = 50)
**Age groups (years)**			
0-10	1	0	1
11-20	1	0	1
21-30	5	0	5
31-40	6	4	10
41-50	6	1	7
51-60	8	5	13
>60	11	2	13
**Gender**			
Male	20	7	27
Female	18	5	23
**Occupation**			
Children/Students	4	0	4
Homemaker	12	3	15
Farmer	6	0	6
Business	2	0	2
Labourer	2	2	4
Retired professionals	2	1	3
Railways	1	0	1
Teacher	2	0	2
Nurse	1	0	1
Unknown	6	6	12

Abbreviations: MABC, *Mycobacterium abscessus* complex.

**Clinical manifestations:** The distribution of infection types differed notably (Table 2). Extrapulmonary infections predominated (32/50, 64.0%) in both subspecies (*M. abscessus* subsp. *abscessus*: 24/38, 63.1%; *M. abscessus* subsp. *massiliense*: 8/12, 66.7%). Pulmonary cases (n = 18) were mainly diagnosed from sputum specimens, followed by bronchoalveolar lavage and gastric lavage. Skin and soft-tissue infections were the most common extrapulmonary manifestation (21/32, 65.6%), particularly among *M. abscessus* infections (17/24, 70.8% of extrapulmonary cases), followed by urinary system infections, whereas bloodstream infections were rare (3/30, 10%).

**Table 2:** Clinical manifestations and sample types of MABC infections.

Category	*M. abscessus* (n = 38)	*M. massiliense* (n = 12)	Total (n = 50)
**Infection type**			
Pulmonary	14	4	18
Extrapulmonary	24	8	32
**Pulmonary samples**			
Sputum/induced sputum	11	4	15
Bronchoalveolar lavage	2	0	2
Gastric lavage	1	0	1
**Extrapulmonary samples**			
Skin and soft tissue	17	4	21
Bloodstream	2	1	3
Urinary system	5	3	8

Abbreviations: MABC, *Mycobacterium abscessus* complex.

**Underlying conditions:** Diverse comorbid conditions were identified among the study participants (Table 3). Previous pulmonary tuberculosis (PTB) and diabetes were the most frequent conditions (12 patients each, 24%). Fungal infections and bronchiectasis were observed in four patients each (8%). Chronic obstructive pulmonary disease, autoimmune disease, HIV, and cancer were infrequent, whereas asthma, CF, and intravenous drug use were absent. Male patients were more frequently affected by chronic obstructive pulmonary disease, fungal disease, bronchiectasis, and diabetes, whereas all HIV and cancer cases occurred in females.

**Table 3:** Underlying conditions.

	COPD	Fungal disease	Cystic fibrosis	Asthma	History of previous TB	Bronchiectasis	HIV	Cancer	IV drug use	Diabetes	Autoimmune disorder
Male	1	3	0	0	6	3	0	0	0	9	1
Female	0	1	0	0	6	1	1	1	0	3	0
**Total**	**1**	**4**	**0**	**0**	**12**	**4**	**1**	**1**	**0**	**12**	**1**
Subsp. *abscessus*	0	3	0	0	10	4	1	1	0	12	0
Subsp. *massiliense*	1	1	0	0	2	0	0	0	0	0	1

Abbreviations: COPD, chronic obstructive pulmonary disease; HIV, human immunodeficiency virus; IV, intravenous; TB, tuberculosis.

**Clinical outcome:** Clinical outcomes were available for a subset of patients included in this study. Among these patients, 12 were cured, indicating successful completion of therapy with clinical improvement. Seven patients were still receiving treatment at the time of data collection. A substantial proportion of patients (n = 29) were lost to follow-up, limiting the long-term assessment of treatment response. In addition, two patients were discharged against medical advice.

Overall, the high rate of loss to follow-up highlights the challenges in managing and monitoring of MABC infections in routine healthcare settings.

**Figure 1:**
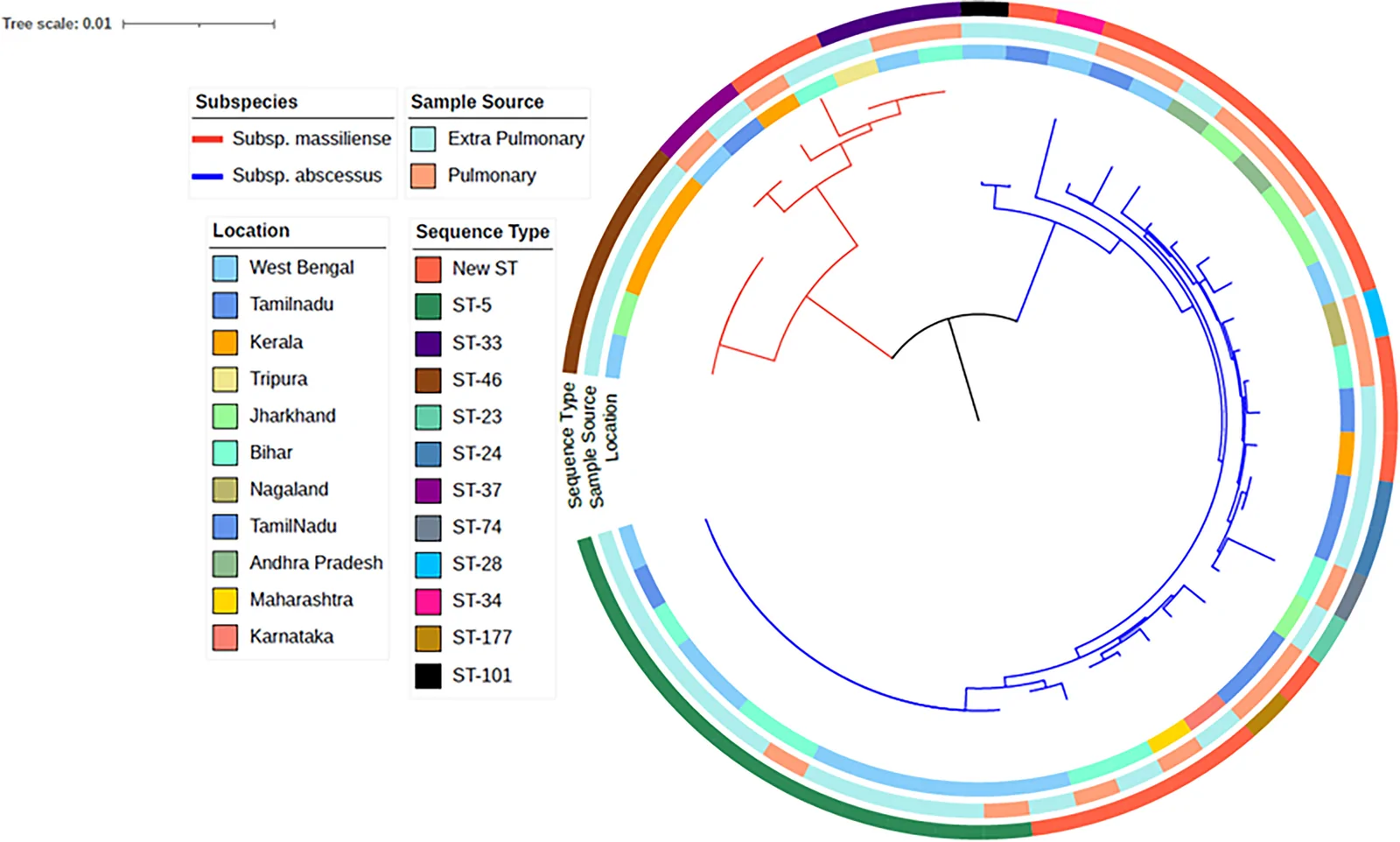
Phylogeny of 50 Indian MABC isolates. The different colors on the branches indicate different subspecies. The inner ring represents the patient location, the middle ring shows the sample source, and the outer ring indicates the STs based on the 7-locus MLST scheme hosted on the PubMLST database.

Abbreviations: MABC, *Mycobacterium abscessus* complex; MLST, multilocus sequence typing; ST, sequence type.

**MLST ST distribution:** MLST analysis of the 50 MABC isolates revealed substantial genetic diversity in India. The distribution of STs by subspecies is detailed in Table 4. Among *M. abscessus* subsp. *abscessus* (n = 38), isolates were distributed across 8 known STs and 18 novel STs. ST-5 was predominant (32.4%, 12/38). ST-24 was the second most commonly known type (5.4%, two of 38). The remaining known STs (ST-23, ST-28, ST-34, ST-74, ST-101, ST-177) and all novel STs (ST-315 to ST-338; 18 types) were each represented by a single isolate. Overall, 48.6% (18/38) of the isolates belonged to novel STs. The sequence type distribution of the MABC isolates is summarized in Table 5. The phylogenetic relationships of the 50 MABC isolates are shown in Fig. 1.

**Table 4:** Clinical outcomes of the patients.

Clinical outcome	No. of patients
Cured	12
On treatment	7
Lost to follow-up	29
Discharged against medical advice	2

**Table 5:** Sequence type distribution of *M. abscessus* complex isolates in India.

Subspecies	ST	No. of strains (%)
***M. abscessus* subsp. *abscessus***	**Known STs:**	
	ST-5	12 (32.4%)
	ST-24	2 (5.4%)
	ST-23, 28, 34, 74, 101, 177	1 each (15.7% total)
	**Novel STs**	
	ST-315, 316, 317, 318, 319, 320, 326, 327, 328, 329, 330, 331, 332, 333, 334, 335, 337, and 338	18 (48.6%)

***M. abscessus* subsp. *massiliense***	**Known STs:**	
	ST-46	5 (41.7%)
	ST-33	3 (25%)
	ST-37	2 (16.7%)
	**Novel STs**	
	ST-314, ST-336	2 (16.7%)

Abbreviation: ST, sequence type.

Among *M. abscessus* subsp. *massiliense* (n = 12), most clustered into ST-46, ST-33, and ST-37, with two novel STs identified. Overall, 40% (20/50) of the isolates represented previously unreported STs, underscoring the substantial genetic diversity in Indian MABC strains.

## Discussion

### Subspecies prevalence

This study outlines the geographic, clinical, and molecular epidemiology of MABC isolates in India. *M. abscessus* subsp. *abscessus* (76%) was the predominant subspecies, followed by *M. abscessus* subsp. *massiliense* (24%), consistent with other Indian and global reports identifying this subspecies as the most common pathogen in both pulmonary and extrapulmonary disease [4,17]. Although biochemical assays are useful for preliminary identification, they do not reliably differentiate subspecies within the MABC. Subspecies-level identification is clinically important because *M. abscessus* subsp. *massiliense* is typically associated with an improved response to macrolide due to a nonfunctional *erm(41)* gene, whereas *M. abscessus* subsp. *abscessus* commonly exhibits inducible macrolide resistance. Therefore, molecular approaches such as MALDI-TOF MS, MLST, and core-genome multilocus sequence typing (cgMLST) are essential for accurate subspecies-level classification and high-resolution epidemiological investigations.

In the present study, both subspecies were more frequently isolated from extrapulmonary sites, in contrast to Western reports, in which *M. abscessus* subsp. *massiliense* is often associated with pulmonary disease in patients with CF. Our findings reveal a higher frequency of extrapulmonary infections in Indian cases, suggesting distinct local epidemiological patterns or host susceptibility differences. The absence of *M. abscessus* subsp. *bolletii* is consistent with previous reports showing that this subspecies is infrequently isolated in clinical settings worldwide.

### Geographic distribution

The predominance of *M. abscessus* subsp. *abscessus*, particularly in West Bengal, Bihar, and Tamil Nadu, suggests either a higher disease burden or a higher proportion of patients seeking medical care at our institution. This finding emphasizes the need for routine culture and species identification of clinically relevant NTMs across India. The regional clustering also aligns with global observations of geographic heterogeneity, where variations in environmental exposure and health care infrastructure contribute to differences in subspecies distribution.

### Clinical manifestations

The comorbidity profile aligns with global evidence regarding host factors that predispose individuals to NTM disease. Most cases had pre-existing pulmonary disease, whereas no patients had CF, consistent with evidence that CF is rare in India [12]. Prior PTB was the most frequent condition, consistent with studies showing that post-TB structural lung damage predisposes individuals to NTM infections [18].

Diabetes mellitus further supports existing evidence that hyperglycemia-associated immune dysfunction heightens susceptibility to mycobacterial infections [19]***.*** The presence of bronchiectasis and chronic fungal disease supports established links between chronic airway inflammation and NTM colonization [20]. Similar patterns have been documented in Indian studies, where PTB-related structural disease, diabetes, and chronic respiratory disorders remain the predominant risk factors for NTM infections [21,22].

### Clinical outcomes and follow-up

Clinical outcome data were available for a subset of patients in this study. Among these patients, 12 patients were cured, indicating a favorable response following completion of therapy. However, seven patients were still receiving treatment at the time of analysis, reflecting the prolonged and complex treatment course typically required for MABC infections. Notably, a large proportion of patients (n = 29) were lost to follow-up, and two patients were discharged against medical advice. A key limitation of this study is that detailed long-term follow-up outcomes were not uniformly available for all patients.

### Sequence Type (ST)

MLST analysis revealed ST-5 as the prevalent ST, suggesting the persistence of a dominant clone or a widely distributed environmental lineage across geographically distant states. Similar cgMLST-based investigations have reported the worldwide circulation of dominant STs, often associated with hospital-linked or pseudo-outbreak clusters [9,23]. The identification of 18 novel STs further highlights the complex genetic landscape of the *M. abscessus* complex in India, emphasizing the need to expand global MLST repositories to better capture diversity from underrepresented regions. In *M. abscessus* subsp. *massiliense*, the predominance of ST-46, ST-33, and ST-37 mirrors findings from international studies [24,25], in which these STs have been linked to both clinical and occasional healthcare-associated clusters. The lower number of divergent STs in *massiliense* may reflect differences in transmission dynamics between subspecies. Together, these findings highlight the value of high-resolution molecular typing tools such as MLST/cgMLST for baseline genomic surveillance and for identifying genetically related isolates that may warrant further epidemiological investigation.

## Conclusion

Infections caused by NTMs are increasing, particularly those caused by the MABC. In this study, *M. abscessus subsp. abscessus* was the predominant subspecies, and MLST analysis identified ST-5 as the most common ST, with multiple STs distributed across geographically distinct regions. The identification of several novel STs further highlights the genetic diversity of MABC isolates circulating in India.

Although the current dataset does not allow direct inference of transmission dynamics, our findings support the utility of MLST and cgMLST as effective tools for molecular characterization and baseline genomic surveillance of MABC. Future studies incorporating broader geographic sampling, correlations with antimicrobial susceptibility, and comprehensive clinical outcome data will be critical for strengthening epidemiological interpretation and improving the clinical relevance of these findings in the Indian context.

## Declaration of competing interest

The authors have no competing interests to declare.
